# The Impact of COVID-19 on Infection Control Measures in Dental Settings: A Cross-Sectional Study

**DOI:** 10.3390/ijerph20156517

**Published:** 2023-08-04

**Authors:** Yasmeen Wahdan, Ghassan Habash, Elham Kateeb, Raed Junaidy, Soher Nagi Jayash

**Affiliations:** 1 Institiute of Community and Public Health, Birzeit University, West Bank, Birzeit P.O. Box 14, Palestine; 2 Faculty of Graduate Studies, Arab American University, Jenin P.O. Box 240, Palestine; 3 Palestinian Association of Dental Implantology, Palestinian Dental Association, Al-Quds P.O. Box 19183, Palestine; 4 Oral Health Research and Promotion Unit, Al-Quds University, Jerusalem P.O. Box 51000, Palestine; 5 Dental Unit, Ministry of Health, Jerusalem P.O. Box 69018, Palestine; 6 The Roslin Institute and Royal (Dick) School of Veterinary Studies, University of Edinburgh, Edinburgh EH25 9RG, UK

**Keywords:** COVID-19, dentists, survey, questionnaires, dental practices, West Bank, pandemics

## Abstract

The COVID-19 pandemic has had a significant impact on every aspect of life, especially for healthcare professionals. Dentists are the most at risk of infection due to close contact with patients. This study aimed to assess the level of awareness, perception, and attitude of Palestinian dentists towards COVID-19 and infection control. A cross-sectional online survey was conducted from 17–30 July 2020, and 349 dentists from the West Bank participated. The survey assessed demographic variables, participation in infection control training, prevention methods used in dental clinics, patient preparation for dental work, cross-infection control and sterilization before and after the pandemic, and sources for guideline protocols for dental workers. The results of the study showed that 54 (14.4%) dentists had received training in infection control in dentistry and 121 (34.3%) had attended training specifically regarding COVID-19. During a partial lockdown, 60% of dentists treated only urgent cases. Overall, the dentists in the West Bank demonstrated good knowledge and a positive attitude towards COVID-19 and infection control measures in dental clinics, as there were significant differences between replacing a medical apron or mask and wearing a face shield, cover shoes, head cap, and goggles before and after COVID (*p* < 0.05). Moreover, there were significant differences between wrapping the chair and using purification devices to disinfect the clinic before and after COVID (*p* < 0.05). However, dentists’ knowledge could be improved by increasing their accessibility to materials and provided training. Dental associations should provide guidelines regularly to dentists during a crisis to inform them of best practices and disease management. In conclusion, dentists need to update their knowledge, continuing education and training to guarantee the proper handling of COVID-19. The study’s findings show the importance of updating infection control protocols and training programs that address the specific needs and challenges faced by dentists in the West Bank.

## 1. Introduction

The COVID-19 pandemic has had a significant impact on societies worldwide, affecting both developing and industrialized countries [1,2]. The virus, which belongs to the coronavirus family, quickly spread across the globe, leading to strict quarantine measures, international travel restrictions, and changes in healthcare practices [3,4,5]. COVID-19 is characterized as a viral respiratory illness, and the transmission route of COVID-19 is via different pathways, including coughing or sneezing or direct contact with an infected person. The disease can be transmitted between people without symptoms, and this characteristic of the disease helps to spread the disease [6]. Hence, more comprehensive countermeasures are needed to address COVID-19 infections and prevent spread [7,8]. Healthcare workers, including dental practitioners, were at a high risk of contracting the virus due to their direct contact with patients and exposure to saliva, blood, and other body fluids during dental procedures [9,10,11]. Dentists work directly with patients in clinics, and they are unable to maintain more than one meter of interpersonal space. As well as the dental instruments and exposure to saliva, blood from the oral cavity, and other body fluids during procedures, there is the contribution to the spread of the infection in dental clinics [10,12]. Because of this pandemic, dentists had to be more careful when dealing with patients in clinics to protect themselves by providing sufficient time between each patient, as well as using the addition of personal protective equipment (PPE) [13,14]. In addition, dentists have to protect the patients by using cross-infection procedures, such as using a high level of sterilization, providing gloves and masks, and using mouthwashes to reduce viral load [15]. Consequently, the dental community had to adapt to new infection by developing infection control protocols to protect dentists and patients from COVID-19 [13,15]. Global infection control protocols have been developed and include how to prepare clinics for the patients and what type of treatments and tools need to be used [16].

In Italy, there is a significant increase in the mortality rate compared to the previous five years for dentists [17], and treatment efforts were prioritized based on the patient’s condition and whether or not they were deemed critical [18]. Meanwhile, the rest of Europe and the world have implemented strict quarantine procedures, including closing places of worship such as mosques and churches and forbidding gatherings in closed places [2,19].

In Palestine, a protocol was developed by the Palestinian Ministry of Health (MOH) based on the guidelines of the World Health Organization to provide the necessary information on infection control and protection from COVID-19. The protocol was applied in May 2020, and dental practices were allowed to reopen for dental care activity under the conditions applied from the MOH protocol standards [20,21]. However, the pandemic’s second wave was beginning to emerge, and dental practitioners had to modify their practices to minimize COVID-19 transmission during dental treatment.

This study aimed to evaluate the level of knowledge and attitudes regarding COVID-19 and infection control among Palestinian dentists in mid-July 2020 and compared their practices before and after the pandemic.

## 2. Materials and Methods

### 2.1. Study Design and Setting

This research is an observational descriptive survey with a quantitative data collection method. The survey was published in selected Facebook groups from 17–30 July 2020. The survey was shared with all eligible participants (*n* = 650) by using the dentistry-associated closed groups on Facebook and by sending the questionnaire to the eligible dentists individually. The questionnaire was developed based on a thorough literature review and international guidelines for infection prevention and control, including protocols from the CDC (Center for Disease Prevention and Control), ADA (American Dental Association), and MOH (Ministry of Health) [17,19,20]. The questionnaire was written in English and translated to Arabic. The survey contained multiple-choice and yes/no questions.

### 2.2. Participants and Sampling

The target population for this study was Palestinian dentists practicing in the West Bank region of the occupied Palestinian territories. The study used a convenient sampling style, with participants recruited via Facebook groups affiliated with the Palestinian Dental Association. Members of these groups worked in various settings, including MOH dental clinics, private dental clinics, and other dental settings in the West Bank. The survey was distributed online using Google Docs.

### 2.3. Sample Size Calculation

The sample size was calculated using RAOSOAFT online sample size calculator (http://www.raosoft.com/samplesize.html (accessed on 6 May 2020)), with a confidence level of 95%, a margin of error of 5%, and a response distribution of 50%. The recommended sample size was 337 dentists, while 349 dentists participated in the study.

### 2.4. Variables and Data Measurement

The survey questionnaire had six sections. The first section collected demographic information about participants, including gender, age, education, specialization, years of practice, working period (full-time or part-time), and workplace. The second section asked about dental procedures provided only in urgent cases and whether participants had received infection control training before or after the pandemic. The third section asked about prevention methods used in dental clinics before and during the COVID-19 pandemic, including the use of protective equipment (masks, gloves, face shield, goggles, head cap, cover shoes, gown), changing lab coat, cleaning dentist’s hands with sanitizer, measuring the temperature of the dentist, washing medical clothes, and using surgical suction. The fourth section asked about procedures performed at the entrance of the clinic, such as asking patients about COVID-19 symptoms such as fever, providing a thermometer, mouthwash, hand sanitizer, mask, and gloves. The fifth section asked about cross-infection control and sterilization methods, including autoclave, sterilization process of dental clinic air, disinfectant used in dental clinic, use of air-conditioning, dental chair wrapping, sterilization of dental instruments and materials such as dental wedges, cotton rolls, and tissues, and sterilization of dental handpiece. The sixth and final section asked about adherence to guidelines when treating patients, methods for updating information, and sources followed to fight against COVID-19 in dental clinics. All aspects of this study were approved by the Al-Quds University Research Ethics Committee. The rate of participation was calculated. The analysis was done between different factors before and after COVID. The data analysis was done by using IBM SPSS Statistics.

### 2.5. Survey Validation

For validation, the survey was peer-reviewed by experts for content validity. All amendments were made according to experts’ comments.

### 2.6. Data Collection

The questionnaire was accompanied by a cover letter explaining the purposes of the study and the confidential use of the information. Data will be collected anonymously, as they did not include personal details, and stored in a password-protected server.

### 2.7. Statistical Methods

The collected data were carefully checked for completeness and accuracy. Descriptive statistics, including frequencies and percentages, were used to summarize the data for quantitative and categorical variables. All statistical analyses were performed using the Statistical Package for the Social Sciences (SPSS) for Windows, version 24.0.

To determine statistical significance, a *p*-value of less than 0.05 was used. Multiple comparisons were performed using the Sidak test.

## 3. Results

### 3.1. Participants Characteristics

We received 349 responses, and the survey respondents’ ages were between 30 and 39 years old (46.7%). Most respondents were males (50.9%), the majority of respondents have worked in dental practice for more than 10 years (43%) with full-time work, and more than 71% of the participants were dental specialists. The demographic characteristics of the respondents are shown in Table 1.

### 3.2. Cross-Infection Training before or after the Pandemic

Most respondents (59.9%) confirmed that their dental practice provided dental treatments only for emergency cases during the COVID-19 pandemic. More than half of respondents (65%) reported not receiving training, and the vast majority (84%) indicated they did not participate in any training about infection control after the pandemic. At the time of the survey, most respondents (90%) indicated thinking that it is necessary to receive training to fight COVID-19 (Table 2).

### 3.3. Prevention Methods Used in Dental Clinics

Regarding prevention methods used in dental clinics before and during COVID-19 (Table 3), the results show that the awareness of dentists in prevention methods increased sharply from 9.6% to 60% in changing the apron between the patients before and after COVID-19 and from 35.0% to 67.9% in replacing the mask between the patients before and after COVID-19. Half of the respondents (49.6%) changed their gown between patients before and after COVID-19. Regarding wearing an N95 mask, the percentage increased from 2.6% to 26% before and after COVID-19. However, 38.4% of dentists who used N95 or KN95 used the same mask more than three times. The percentages of using a face shield, shoe cover, head cap, and powder-free gloves were 86%, 47%, 66%, and 77.7%, respectively, before COVID-19 compared to 18.6%, 7.4%, 22%, and 77.7% after COVID-19. Regarding hand cleaning, over half of the dentists (61.3%) cleaned their hands with soap and water, and few used sanitizer before COVID-19 (7.7%), while 44% of dentists washed their hands followed by using sanitizer after COVID-19. For wearing goggles, 41% of dentists used them before COVID-19, compared to 79% after COVID-19. Moreover, the majority of dentists (82%) washed medical clothes more than once using high temperatures after COVID-19.

Interestingly, there were significant differences between replacing a medical apron, mask, wearing a face shield, shoes cover, head cap, and goggles before and after COVID (*p* < 0.05).

### 3.4. Patient’s Preparations for Dental Work

In terms of the dentist’s level of preparation for dental work after COVID-19, most dentists (80.5%) asked the patients about COVID-19 symptoms and over half of the respondents used thermometers and asked the patients to rinse their mouth with a mouthwash (57.6%, 75.4%, respectively). There was a significant difference between asking the patient to rinse their mouth with anti-bacterial mouthwash before and after COVID (*p* < 0.05). The most used mouthwash was chlorhexidine before and after COVID-19 (75.4% and 67%, respectively), while H_2_O_2_ was used by 19.2% after COVID-19 compared to 8.6% before COVID-19, and only 5.2% of dentists used povidone-iodine as a mouthwash after COVID-19. Most dentists (85%) provided hand sanitizer, masks, and gloves at the entrance of the clinic for the patient. Table 4 shows the results of dentists’ procedures related to patients.

### 3.5. Cross-Infection Control and Sterilization Process

In the fifth section of the survey (Table 5), the results showed the awareness of the dentists for cross-infection control and sterilization. The percentages of preparation of clinic between patients (20 min), wrapping the dental chair, and autoclaving the handpiece increased markedly from 14.6% to 73.4%, 43.6% to 65.3%, and 60.5% to 79.1% before and after COVID-19, respectively. Most dentists used sodium hypochlorite to disinfect the clinic, and most of them did not use any of the purification devices to disinfect the clinic. For sterilization of dental materials, dentists sterilize dental wedges, tissue, and cotton roll by autoclave after COVID-19 (76%, 43%, and 35%, respectively). Approximately 33% of dentists turn off the AC in the clinic while they are working. There were significant differences between wrapping the chair and using purification devices to disinfect the clinic before and after COVID (*p* < 0.05).

### 3.6. Guidelines and the Information Sources

Finally, in the sixth section of the survey (Table 6), the data showed that most dentists (65%) followed the MoH and the Palestinian Dental Association protocols in the treatment of the patients. The dentists had dental assistants before and after COVID-19 (65% and 60%, respectively). The main source for infection control was the MoH protocol (59%), followed by the WHO protocol (33.8%).

## 4. Discussion

The present study aimed to evaluate the infection control practices adopted by Palestinian dentists to minimize COVID-19 transmission during dental treatment in the West Bank region. For this purpose, a questionnaire focused on the practice and modifications of dental procedures used by dentists to minimize the spreading of COVID-19 and to protect themselves and their patients. The findings showed that most dentists had taken measures to prevent COVID-19 transmission in their clinics, such as using personal protective equipment (PPE), screening patients, and implementing infection control protocols. Most of them replaced facial masks (68%), despite this being a usual procedure even before COVID-19, whereas the use of face shields, head caps, cover shoes, and goggles was high after COVID-19 compared to the use before COVID-19. This result showed significant differences between replacing a medical apron or mask and wearing a face shield, cover shoes, head cap, and goggles before and after COVID (*p* < 0.05). Additionally, there was a significant difference between using anti-bacterial mouthwash before and after COVID (*p* < 0.05). This result showed generally good awareness and knowledge of COVID-19 among the majority of the participants, suggesting the MoH protocol is the most appropriate health protocol that we encourage dentists to follow [7]. About 34% of the participants need 20 min to prepare for the clinic, and this, according to the WHO protocol, is a good time to preserve proper ventilation. These results are consistent with previous studies that have shown that dental professionals have been highly aware of the potential transmission risks of COVID-19 and have implemented measures to mitigate these risks [20,22,23,24].

The present study found that a majority of dentists reported providing only urgent dental care during the pandemic, and this was reported in the previous study [20]. In addition, this approach is in line with international recommendations to limit non-urgent dental procedures to reduce the risk of COVID-19 transmission [16]. Dental treatment limitation to emergencies is important to limit the COVID-19 outbreak [15], and the Palestinian Ministry of Health protocol instructed dental practitioners to treat only urgent cases, and this is in line with the Centers for Disease Control and Prevention (CDC) protocol [16]. All of the prevention measures—ranging from hand washing to protective equipment, including surgical masks, face shields, gowns, and gloves—have been emphasized repeatedly, and this is even more important in the case of dental practitioners [23,25]. Moreover, the present study found that dentists have significantly increased their use of PPE, such as masks, gloves, face shields, goggles, and gowns, during dental procedures. This is a crucial step to protect both patients and healthcare workers from COVID-19 infection, as previous studies have shown that the use of appropriate PPE is associated with a reduced risk of COVID-19 transmission [22].

The present study also found that most dentists have implemented additional infection control measures, such as disinfection of surfaces and instruments, sterilization of dental equipment, and enhanced ventilation in their clinics. This confirms the awareness of dentists, as there were statistically significance differences between wrapping the chair and using purification devices to disinfect the clinic before and after COVID (*p* < 0.05). These infection control procedures are an effective way to control cross-infections, according to the protocol to limit the spread of aerosols on dental surfaces [26]. Before the beginning of the treatment, a third of the respondents turned off the air conditioning during work, and this improved the ventilation of the dental clinic, thus ensuring air movement in appropriate ways and flowing directions according to CDC considerations [16,27].

These findings indicate that dental professionals are taking proactive measures to reduce the risk of COVID-19 transmission in their clinics. However, the study also found that some dentists were not following all recommended infection control practices, such as changing lab coats and cleaning their hands with sanitizer. This highlights the need for continued education and training for dental professionals to ensure that they adhere to infection control protocols and minimize the risk of COVID-19 transmission in their clinics [28].

Interestingly, the present study found that dentists who received infection control training during the pandemic were more likely to adopt infection control practices than those who did not receive training. This highlights the importance of continuing education for dental professionals, particularly during the pandemic, to ensure that they are aware of the latest infection control protocols and guidelines. Dental associations and organizations should continue to provide ongoing education and training for their members to ensure that they are equipped to handle the challenges of the pandemic.

In general, dentists should be aware of the critical COVID-19 situation and should comply with the standard measures needed to improve infection control strategies during this epidemic. Moreover, according to the result of this study, there is a need to continuously update dentists regarding working in pandemics and continuously train and educate the dentists to protect themselves and their patients during pandemics [29,30].

The results of this study could help dentists protect themselves and their patients from the spread of COVID-19 and improve future infection control practices in dental settings during pandemics.

The present study has several limitations. First, the study relied on self-reported data, which may be subject to recall bias and social desirability bias. Second, the study used a convenience sampling method, which may not be representative of all Palestinian dentists in the West Bank region. Therefore, caution should be exercised when generalizing the results of this study to the entire population of Palestinian dentists in the region. Third, the study did not assess the impact of COVID-19 on the dental practice or the financial impact of the pandemic on dental clinics.

## 5. Conclusions

The findings suggest that most dentists have taken measures to prevent COVID-19 transmission in their clinics, but there is still room for improvement, particularly with regard to adherence to infection control protocols. Continued education and training for dental professionals, as well as ongoing monitoring and evaluation of infection control practices, are essential to minimize the risk of COVID-19 transmission in dental clinics. Further research is needed to assess the long-term impact of the pandemic on dental practices and the financial impact on dental clinics.

## Figures and Tables

**Table 1:** Demographic information of dental care professionals in the West Bank.

Demographics	Number (%)
Gender	
Male	178(51%)
Female	171(49%)
Age group	
Less than 30 years	118(33.8%)
30–39 years	163(46.7%)
40–49 years	43(12.3%)
More than 50 years	25(7.2%)
Number of years in dental practice	
Less than 5	154(50.9%)
6–10 years	135(40.1%)
More than 10	60(7.0%)
Current work time	
Full-time	177(50.7%)
Part-time	172(49.3%)
Obtained a master’s degree or residency program in any of the dental specialties	
Yes	101(28.9%)
No	248(71.1%)
Workplace	
Private clinic	314(90.0%)
University dentistry department	18(5.2%)
MOH clinic	7(2.0%)
Others	11(3.1%)

**Table 2:** Information regarding participating in cross-infection training before or after the pandemic.

Question	Number (%)
After the COVID-19 pandemic, do you treat just necessary cases or all cases?	
Necessary cases	201(57.6%)
All cases	148(42.4%)
Have you had any training in infection control in dentistry about COVID-19?	
Yes	121(34.3%)
No	228(65.7%)
Have you participated in any training about COVID-19?	
Yes	54(15.4%)
No	295(84.5%)
Do you think it is necessary to receive training to fight COVID-19?	
Yes	317(90.8%)
No	32(9.2%)

**Table 3:** Prevention methods used in dental clinics before and during COVID-19.

Question	Number (%)
Were you replacing a medical apron between patients before COVID-19?	
Yes	32(9.2%)
No	317(90.8%)
Were you replacing a medical apron between patients after COVID-19?	
Yes	209(59.9%) *
No	140(40.1%)
Do you wear the COVID-19 suit (gown) between patients?	
Yes	173(49.6%)
No	176(50.4%)
Were you replacing the mask between patients before COVID-19?	
Yes	122(35.0%)
No	227(65.0%)
Were you replacing the mask between each patient after COVID-19?	
Yes	237(67.9%) *
No	112(32.1%)
What types of masks did you use before COVID-19?	
Surgical masks	207(59.3%)
N95	9(2.6%)
FFP2	1(0.3%)
3 layer masks	130(37.2%)
What kind of masks do you use after COVID-19?	
Surgical masks	105(30.3%)
N95	92(26.5%)
KN95	41(11.8%)
3 layer masks	75(21.6%)
KN95 with valve	28(8.1%)
FFP2	6(1.7%)
If you use KN95 or N95 after COVID-19, how often do you use it?	
Once	115(33.0%)
Twice	44(12.6%)
Thrice	56(16.1%)
More than thrice	134(38.4%)
Were you wearing a face shield before COVID-19?	
Yes	65(18.6%)
No	284(81.4%)
Were you wearing a face shield after COVID-19?	
Yes	301(86.2%) *
No	48(13.8%)
Did you use cover shoes before COVID-19?	
Yes	26(7.4%)
No	323(92.6%)
Did you use cover shoes after COVID-19?	
Yes	165(47.3%) *
No	184(52.7%)
Did you use a head cap before COVID-19?	
Yes	79(22.6%)
No	270(77.4%)
Did you use a head cap after COVID-19?	
Yes	233(66.8%) *
No	116(33.2%)
What kind of gloves did you use before COVID-19?	
Powder-free	271(77.7%)
With latex	78(22.3%)
What kind of gloves did you use after COVID-19?	
Powder-free	271(77.7%)
With latex	78(22.3%)
How to clean your hands before wearing gloves before COVID-19?	
Use hand sanitizer	47(13.5%)
Use soap and water	214(61.3%)
Wearing gloves without hand cleaning	27(7.7%)
Use soap and water and sanitizer	61(17.5%)
How to clean your hands before wearing gloves after COVID-19?	
Use hand sanitizer	68(19.5%)
Use soap and water	125(35.8%)
Wearing gloves without hand cleaning	6(1.7%)
Use soap and water and sanitizer	150(43.0%)
Were you wearing goggles before COVID-19?	
Yes	145(41.5%)
No	204(58.5%)
Were you wearing goggles after COVID-19?	
Yes	276(79.1%) *
No	73(20.9%)
Do you check your temperature and the crew every morning after COVID-19?	
Yes	155(44.4%)
No	194(55.6%)
Do you wash medical clothes more than once using high temperatures after COVID-19?	
Yes	288(82.5%)
No	61(17.5%)
Do you use high-volume suction in your practice after COVID-19?	
Yes	235(67.3%)
No	114(32.7%)

* indicate the significantly different results before and after COVID (*p* < 0.05) using multiple comparisons with the Sidak test.

**Table 4:** The dentists’ procedures related to patient’s preparations for dental work.

Questions	Numbers (%)
Do you take information from patients about the symptoms related to COVID-19 or contact COVID-19 patients before visiting the clinic?	
Yes	281(80.5%)
No	68(19.5%)
Did you use a thermometer after COVID-19?	
Yes	201(57.6%)
No	148(42.4%)
Did you ask every patient to rinse his/her mouth with anti-bacterial mouthwash before treatment before COVID-19?	
Yes	157(45.0%)
No	192(55.0%)
Did you ask every patient to rinse his/her mouth with an anti-bacterial mouthwash before treatment after COVID-19?	
Yes	263(75.4%) *
No	86(24.6%)
Which of the mouthwashes were you using before starting the treatment before COVID-19?	
Chlorhexidine	263(75.4%)
H_2_O_2_	30(8.6%)
Listerine	34(9.7%)
Povidone-iodine	5(1.4)
Other (mouthwashes with polyvinylpyrrolidone/menthol/thymol)	3(0.9%)
Nothing	14(4.0%)
Which of the mouthwashes were you using before starting the treatment after COVID-19?	
Chlorhexidine	234(67.1%)
H_2_O_2_	67(19.2%)
Listerine	15(4.3%)
Povidone-iodine	18(5.2%)
Other (mouthwashes with polyvinylpyrrolidone/menthol/thymol)	14(4.0%)
Nothing	1(0.3)
Did you provide hand sanitizer, mask, and gloves at the entrance of the clinic?	
Yes	300(86.0%)
No	49(14.0%)

* indicate the significantly different results before and after COVID (*p* < 0.05) using multiple comparisons with the Sidak test.

**Table 5:** Cross-infection control and sterilization process.

Questions	Numbers (%)
Before COVID-19, how long did you take to prepare the clinic for the patient?	
10 min	256(73.4%)
20 min	51(14.6%)
30 min	24(6.9%)
More than 30 min	18(5.2%)
After COVID-19, how long do you take to prepare the clinic for the patient?	
10 min	117(33.5%)
20 min	121(34.7%)
30 min	62(17.8%)
More than 30 min	49(14.0%)
Did you wrap the chair before the COVID-19?	
Yes	152(43.6%)
No	197(56.4%)
Did you wrap the chair after the COVID-19?	
Yes	228(65.3%) *
No	121(34.7%)
Did you use an autoclave to sterilize the handpiece between patients before COVID-19?	
Yes	211(60.5%)
No	138(39.5%)
Did you use an autoclave to sterilize the handpiece between patients after COVID-19?	
Yes	276(79.1%)
No	73(20.9%)
Did you use oxygen gas or other gases or any of the purification devices to disinfect the clinic before COVID-19?	
Yes	29(8.3%)
No	320(91.7%)
Did you use oxygen gas or other gases or any of the purification devices to disinfect the clinic after COVID-19?	
Yes	92(26.4%) *
No	257(73.6%)
What was used to disinfect the clinic floor before COVID-19?	
Sodium hypochlorite	186(53.3%)
Cleaning supplies	129(37.0%)
H_2_O_2_	18(5.2%)
Other	16(4.6%)
What did you use to disinfect the clinic floor after COVID-19?	
Sodium hypochlorite	241(69.1%)
Cleaning supplies	48(13.8%)
H_2_O_2_	39(11.2%)
Other	21(6.0%)
Did you sterilize the dental wedges and matrices before COVID-19?	
Yes	220(63.0%)
No	129(37.0%)
Did you sterilize the dental wedges and matrices after COVID-19?	
Yes	266(76.2%)
No	83(23.8%)
Did you sterilize tissues by autoclaving before COVID-19?	
Yes	132(37.8%)
No	217(62.2%)
Did you sterilize tissues by autoclaving after COVID-19?	
Yes	151(43.3%)
No	198(56.7%)
Did you sterilize the cotton rolls by autoclave before COVID-19?	
Yes	94(26.9%)
No	255(73.1%)
Did you sterilize the cotton rolls by autoclave after COVID-19?	
Yes	122(35.0%)
No	227(65.0%)
Do you turn off the air conditioner while working after COVID-19?	
Yes	114(32.7%)
No	235(67.3%)

* indicate the significantly different results before and after COVID (*p* < 0.05) using multiple comparisons with the Sidak test.

**Table 6:** Data related to following the guidelines and the information sources regarding dental treatments during COVID-19.

Questions	Number (%)
Did you follow the MoH and Palestinian Dental Association protocol during COVID-19?	
Yes	228(65.3%)
No	121(34.7%)
What is the main source to update infection control procedures in the clinic?	
MoH protocol	207(59.3%)
WHO protocol	118(33.8%)
ADA protocol	10(2.9%)
CDC protocol	6(1.7%)
Other	8(2.3)
Did you have an assistant in the clinic before COVID-19?	
Yes	228(65.3%)
No	121(34.7%)
Did you have an assistant in the clinic after COVID-19?	
Yes	208(59.6%)
No	141(40.4%)

## Data Availability

Data is contained within the article.
